# Effect of UV/ozone treatment on polystyrene dielectric and its application on organic field-effect transistors

**DOI:** 10.1186/1556-276X-9-479

**Published:** 2014-09-10

**Authors:** Wei Huang, Huidong Fan, Xinming Zhuang, Junsheng Yu

**Affiliations:** 1 State Key Laboratory of Electronic Thin Films and Integrated Devices, School of Optoelectronic Information, University of Electronic Science and Technology of China (UESTC), Chengdu 610054, People's Republic of China

**Keywords:** Polystyrene dielectric, UV/ozone treatment, X-ray photoelectron spectroscopy, Organic field-effect transistor (OFET)

## Abstract

The influence of UV/ozone treatment on the property of polystyrene (PS) dielectric surface was investigated, and pentacene organic field-effect transistors (OFETs) based on the treated dielectric was fabricated. The dielectric and pentacene active layers were characterized by atomic force microscopy, X-ray photoelectron spectroscopy, and scanning electron microscopy. The results showed that, at short UVO exposure time (<10 s), the chemical composition of PS dielectric surface remained the same. While at long UVO exposure time (>60 s), new chemical groups, including alcohol/ether, carbonyl, and carboxyl/ester groups, were formed. By adjusting the UVO exposure time to 5 s, the hole mobility of the OFETs increased to 0.52 cm^2^/Vs, and the threshold voltage was positively shifted to -12 V. While the time of UVO treatment exceeded 30 s, the mobility started to shrink, and the off-current was enlarged. These results indicate that, as a simple surface treatment method, UVO treatment could quantitatively modulate the property of PS dielectric surface by controlling the exposure time, and thus, pioneered a new way to modulate the characteristics of organic electronic devices.

## Background

Due to the low fabrication costs and wide applications, such as large area sensor arrays, flat panel displays, and radio frequency identification tags, organic field-effect transistors (OFETs) as an emerging kind of organic electronic device have been extensively researched in the past few decades [1-3]. As one of the most important components of OFETs, dielectric plays a key role in adjusting the performance of the devices [4-7]. Especially, since the dielectric consisting of polymer possesses excellent bendable feature, which is crucial in flexible nanoscale electronics, this kind of material has been widely studied [8-11].

Moreover, as the conducting channel lies in the first few semiconducting layers in proximity to the gate dielectric, the property of dielectric/organic semiconductor interface at nanoscale will dramatically influence the performance of the OFETs [12,13]. Therefore, the polymer dielectric surface holds the capability to modulate the electronic properties of the devices [14,15], and many methods have been applied to modify the surface [16-18]. Among various strategies, inserting buffer layer is the most common way to modify the dielectric surface. Many materials, including self-assembled monolayer [19,20], organic semiconductor [21,22], and polymer insulator [23], can act as an efficient buffer by improving the dielectric/organic semiconductor interface property. However, inserting buffer to the interface requires additional functional materials, and causes more complicated device architecture, leading to cost increase.

On the other hand, according to previous works on polymers with UV/ozone (UVO) treatment, an obvious change on the surface chemical structure of polystyrene (PS) was observed after UVO treatment [24-26]. Hence, OFETs with UVO-treated dielectric have been studied and exhibited obvious performance variation. However, the specific change of UVO-treated PS dielectric at nanoscale size and its effect on the performance of OFETs have seldom been investigated.

In this work, we studied the influence of UV/ozone treatment on the property of PS dielectric surface in detail. The morphologies of PS dielectric and pentacene layers were characterized by atomic force microscopy (AFM) and scanning electron microscopy (SEM), respectively. And the property of PS dielectric with various UVO treating time was studied by analyzing the PS through X-ray photoelectron spectroscopy (XPS) and electronic characterization. Furthermore, OFETs based on UVO-treated PS dielectrics were fabricated, and the performance change of the OFETs was studied.

## Methods

### Field-effect transistor fabrication

**Figure 1:**
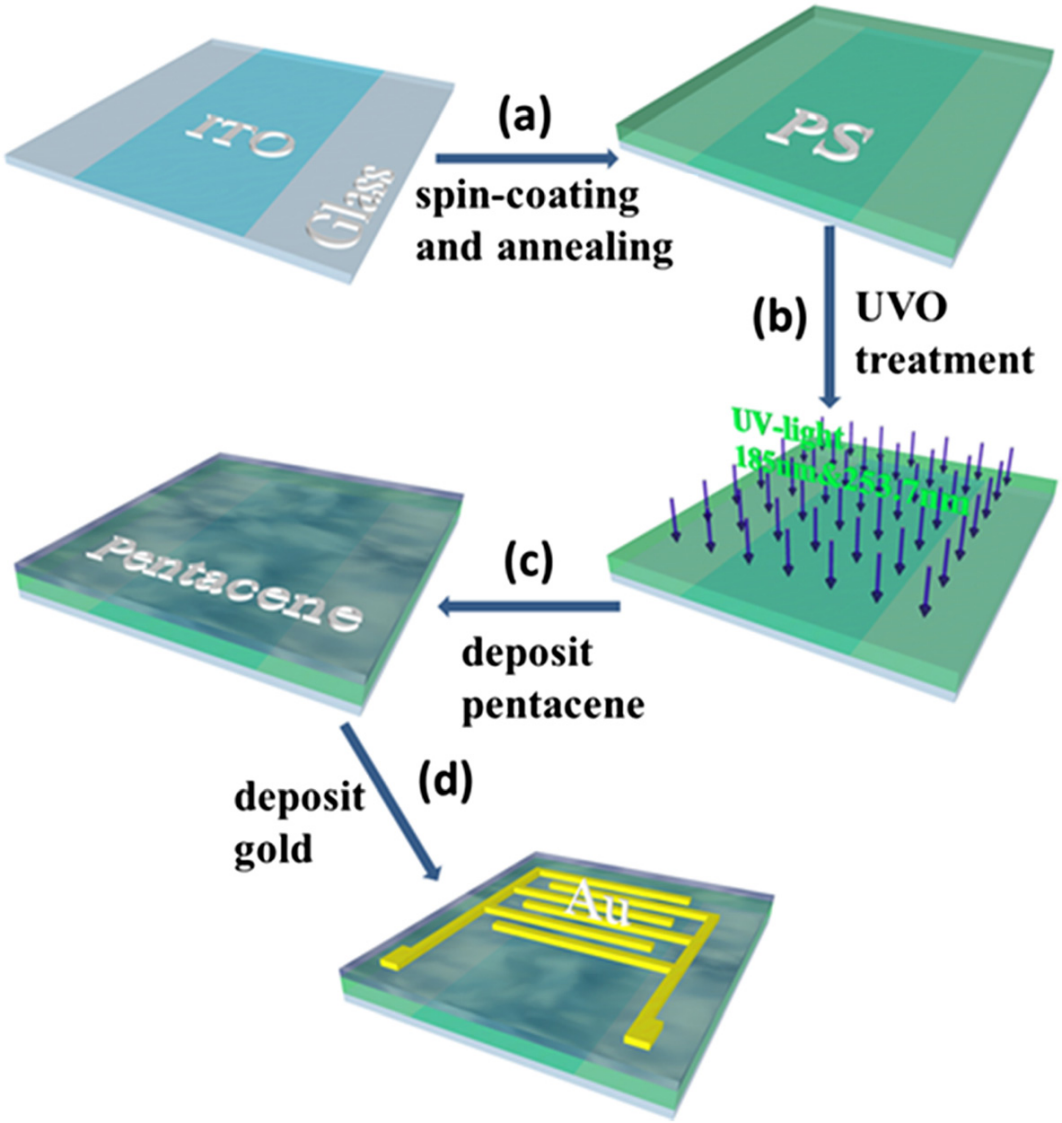
Schematic of the fabrication process of OFETs.

Figure 1 shows the device fabrication process, along with the device architecture. PS (average Mw ~ 280,000) and pentacene were purchased from Sigma-Aldrich (Sigma-Aldrich, St. Louis, MO, USA) and TCI (TCI, Tokyo, Japan), respectively. Indium tin oxide (ITO) glass substrate was cleaned in acetone, deionized water, and isopropyl alcohol for 15 min each by an ultrasonic bath sequentially. A 540 nm PS dielectric was formed by spin-coating a solution of PS in xylene (6 wt.%) on the substrate at room temperature. Then, the dielectric layer was baked at 120°C for 1 hr to completely remove residual solvents. Prior to the deposition of pentacene, the devices were exposed to UV light of 185 and 253.7 nm (SunMonde, UV-O_3_ Cleaner, 40 W, Shanghai, China) for 0 ~ 240 s. Then, pentacene was evaporated under 3 × 10^-4^ Pa at a rate of 0.2 ~ 0.3 Å/s to form a film with 30 nm. At last, gold source and drain electrodes of 50 nm were thermal evaporated using a metal shadow mask without breaking the vacuum.

### Thin film and device characterization

The morphology of PS dielectrics and pentacene films were characterized by AFM (MFP-3D-BIO, Asylum Research, Santa Barbara, CA, USA) in tapping mode and SEM (Hitachi, S-4800, Chiyoda-ku, Japan), respectively. XPS (Omicron ESCA Probe, Tokyo, Japan) characterization of carbon 1 s signal was monitored on PS dielectrics. The capacitance of the gate dielectrics was obtained by measuring capacitance-frequency properties of ITO/PS/Au with Agilent 4294A (Santa Clara, CA, USA).

The electrical characteristics of the OFETs were measured using a Keithley 4200 (Keithley, Cleveland, OH, USA) in a N_2_-filled glove box. The mobility (*μ*) was calculated in the saturation region of transfer curves using (1):

(1) $$I_{\mathrm{ds}}=\left(W/2L\right)\mu C_{i}{\left(V_{\mathrm{gs}}-V_{\mathrm{th}}\right)}^{2}$$

where *L* (100 μm) and *W* (1 cm) were the channel length and channel width, respectively. *C*_i_ was the capacitance (per unit area) of the dielectric, *V*_gs_ was the gate voltage, and *I*_ds_ was the drain-source current.

## Results and discussion

**Figure 2:**
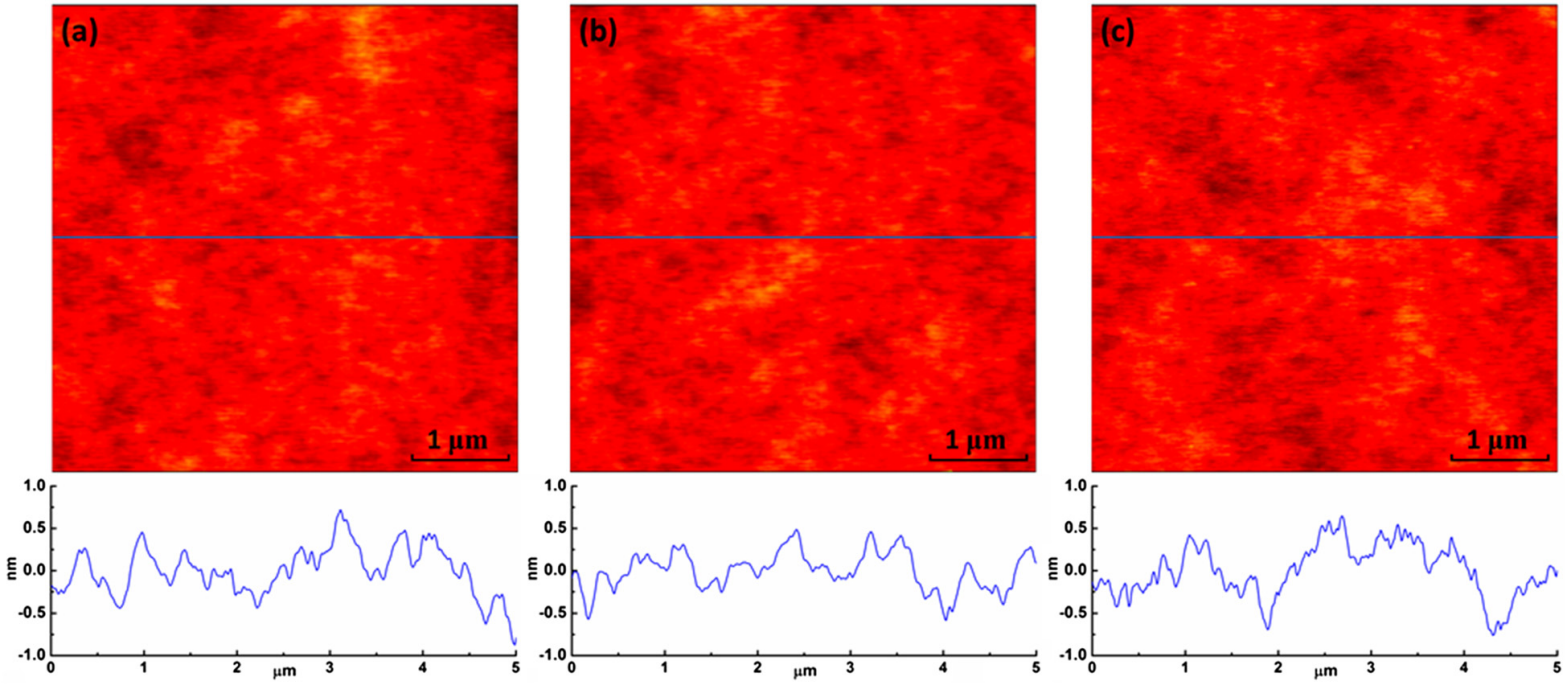
**AFM topography images.** AFM topography images along with the cross-sections of untreated PS **(a)**, 60-s UVO-treated PS **(b)**, and 240-s UVO-treated PS **(c)**, respectively.

Surface roughness is one of the most important properties of dielectric layer, for smooth surface will facilitate the better formation of upper film. Thus, the surface of PS dielectric with/without UVO treatment was analyzed through AFM, as shown in Figure 2. Flat surface was obtained in all the devices, with root mean square roughness of 270 pm on untreated PS, 257 pm on 60-s UVO-treated PS, and 274 pm on 240-s UVO-treated PS. Similar morphologies of the dielectrics implied that UVO treatment would not affect the surface roughness of PS dielectric.

**Figure 3:**
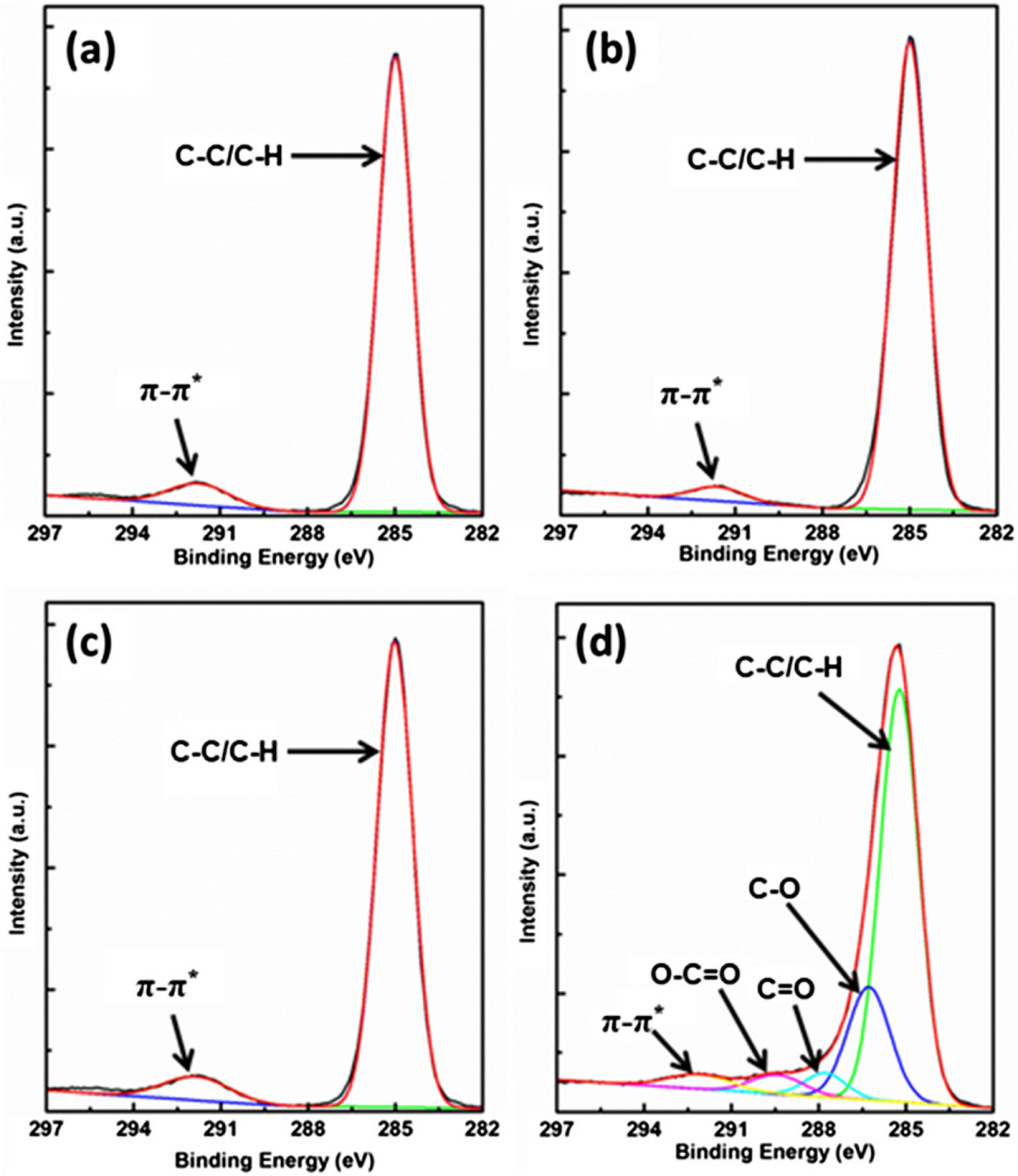
**High-resolution carbon 1 s spectra.** High-resolution carbon 1 s spectra of PS films with fitted curves: **(a)** untreated, **(b)** 5 s UVO treatment, **(c)** 10 s UVO treatment, and **(d)** 60 s UVO treatment.

XPS was also utilized to analyze the surface chemistry of PS dielectrics with/without UVO treatment. As shown in Figure 3a, the carbon 1 s spectrum of the untreated film matched that of pure PS very well, i.e., a hydrocarbon C-C/C-H peak at 285 eV and π-π* shake-up satellites at about 291.5 eV from hydrocarbon peak. Moreover, the spectra of 5- and 10-s UVO-treated PS dielectrics show the same peaks as that of untreated film. At long UVO treatment time (60 s), the formation of new function groups was observed, as shown in Figure 3d. The envelopes of the 60-s UVO-treated film could be resolved into four components: a main hydrocarbon peak at 285 eV, and three peaks corresponding to alcohol/ether C-O (286.5 eV), carbonyl C = O (288 eV), and carboxyl/ester O-C = O (289.5 eV) [25-27]. Therefore, short time UVO treatment would not effectively change the chemical composition of PS dielectric surface. When the exposure time reached to 60 s, an obvious proliferation of additional oxygen functional groups on UVO-treated PS surface could be detected.

**Figure 4:**
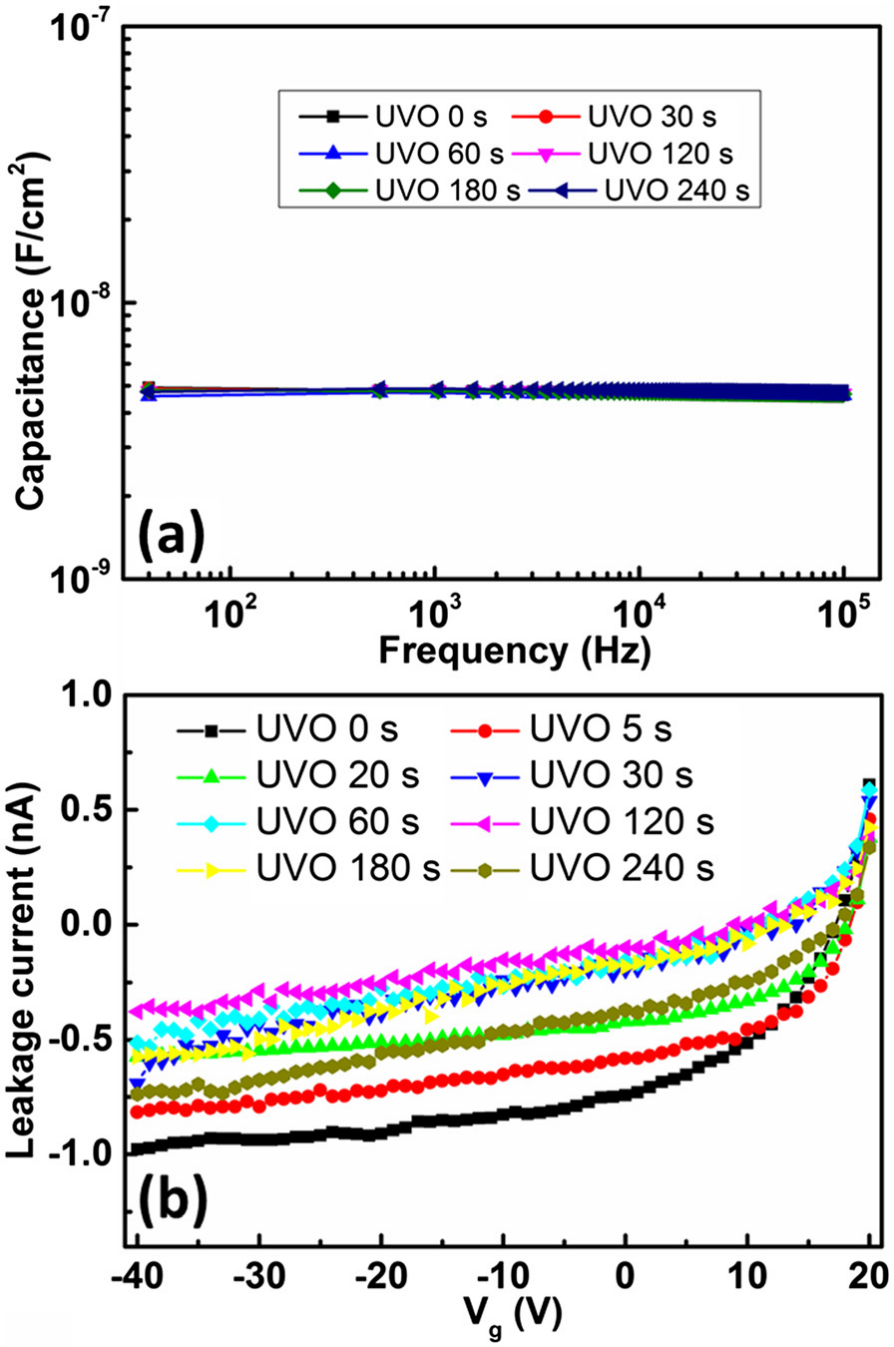
**Capacitance-frequency characteristics and leakage current. (a)** Capacitance-frequency characteristics of ITO/PS/Au structure. **(b)** Leakage current of UVO-treated PS dielectrics with various UVO treatment time.

**Figure 5:**
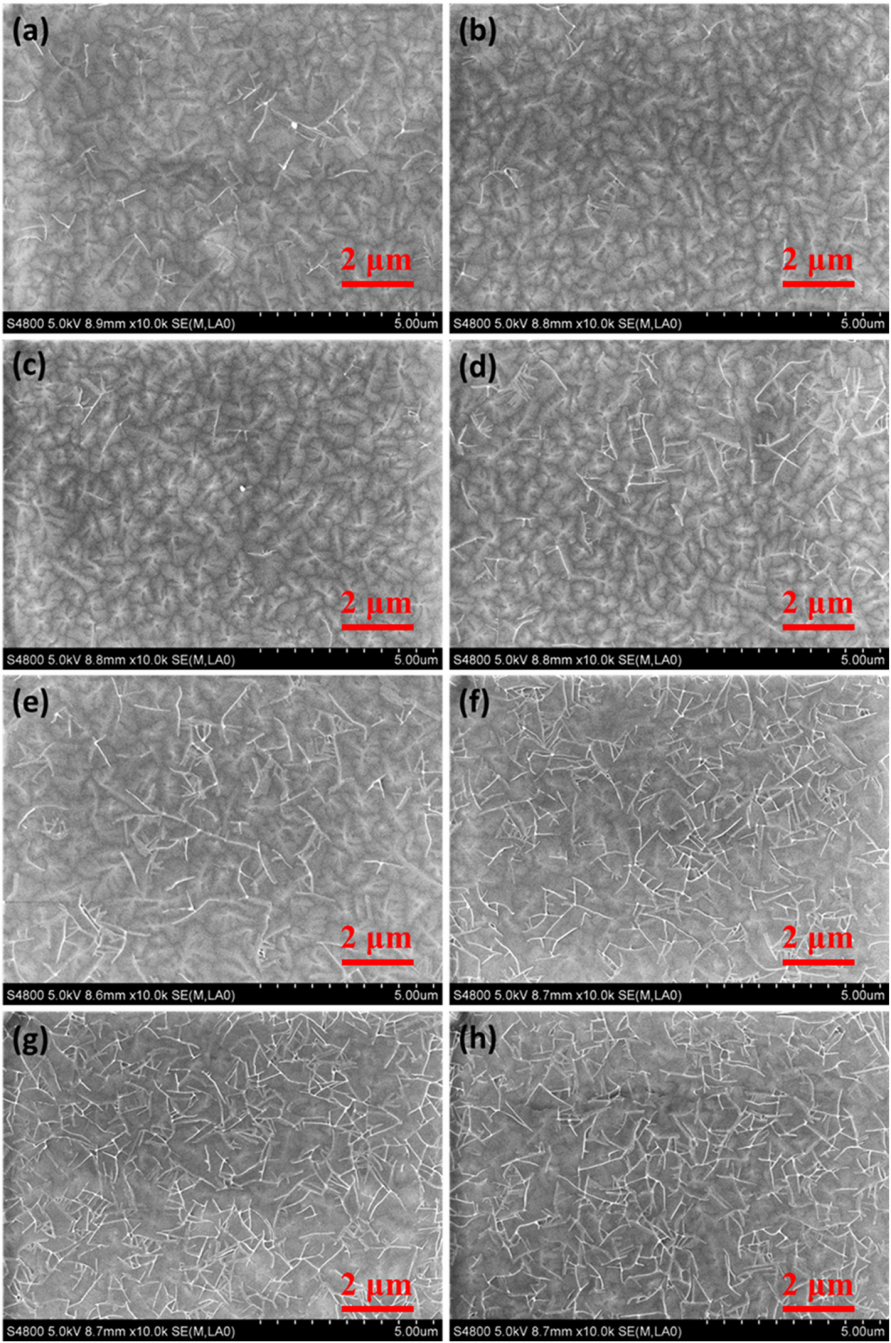
**SEM images.** SEM images of pentacene (30 nm) on PS dielectric with **(a)** 0, **(b)** 10, **(c)** 20, **(d)** 30, **(e)** 60, **(f)** 120, **(g)** 180, and **(h)** 240 s UVO treatment.

Furthermore, to verify the insulating proper, the capacitance-frequency curves of ITO/PS/Au structure were tested. As shown in Figure 4a, all the dielectric with various UVO exposure time show similar capacitance of approximately 4.5 nF/cm^2^ at 100 kHz. Leakage currents of the ITO/PS/Au structure were also tested, and all the leakage currents kept at a relatively low level of 10^-10^ ~ 10^-9^ A, as shown in Figure 4b. Hence, the capacitance and the insulating property would not be damaged by UVO treatment.To verify if the UVO-treated PS dielectric would affect the growth of upper film, the morphologies of pentacene (30 nm) layers grown on PS dielectrics with various UVO exposure time were investigated through SEM. As shown in Figure 5, well-ordered island shape grains were formed on all the PS dielectrics. Interestingly, it could be found that, the pentacene films intended to maintain the similar grain size and crystal shape when UVO treatment time was controlled to be less than 60 s. When UVO treatment time exceeded 120 s, some strip-like pentacene was observed on the top of the original grain of pentacene. These strips tremendously enlarged the surface roughness, leading to an inferior morphology of pentacene film. Such results indicated that short time (<60 s) UVO-treated PS dielectric would not have significant influence on the morphology of pentacene. While long time (>120 s) UVO-treated PS would effectively affect the film forming property, leading to inferior morphology.

**Figure 6:**
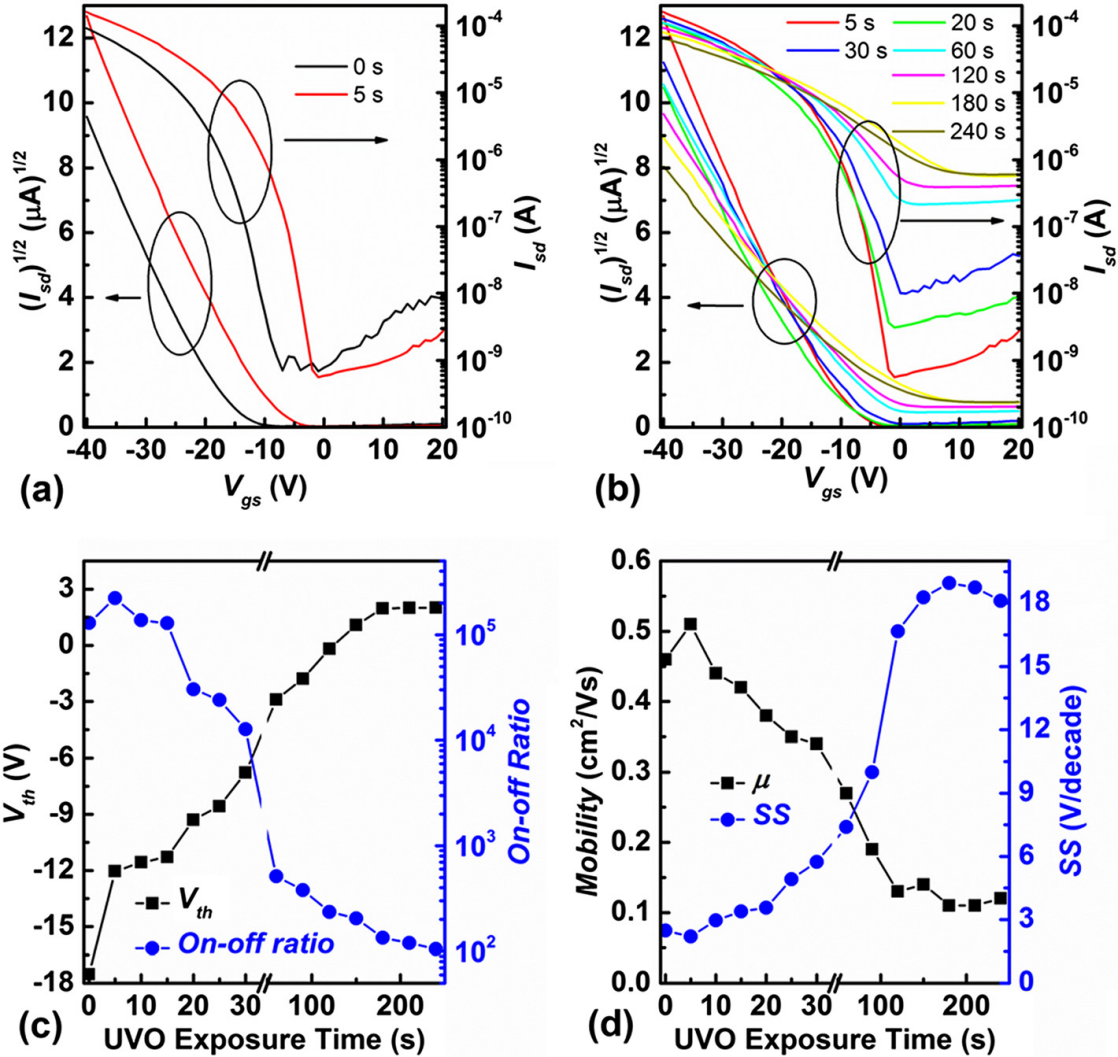
**Transfer curves, *V***_**th**_**, on/off ratio*, μ*, and SS*. *(a) (b)** Transfer curves of pentacene OFETs based on PS dielectrics with UVO treatment time ranging from 0 ~ 240 s, **(c)***V*_th_ and on/off ratio of the pentacene OFETs based on UVO-PS dielectrics as a function of exposure time, and **(d)***μ* and SS of the pentacene OFETs based on UVO-PS dielectrics as a function of exposure time.

Thus, we can see that UVO treatment to PS dielectric could effectively modify the surface functional groups of the dielectric and influence the morphology of the film grown on such dielectric at long exposure time, while no damage to the surface roughness or insulating property were brought. Therefore, OFETs based on UVO-treated PS dielectrics were fabricated, and the electronic characteristics were analyzed. The transfer curves of the devices with 0 ~ 240 s UVO treatment were shown in Figure 6a,b. Also, an additional figure file showed the typical output curves [see Additional file 1]. It is very obvious that UVO treatment on PS dielectric has tremendous influence on the electronic properties of OFETS, including *V*_th_ and off current (*I*_off_). The transistor parameters, i. e., *V*_th_, current on/off ratio (on/off), *μ*, and subthreshold slope (SS), were extracted from the transfer curves, as shown in Figure 6c,d.

Especially, when the UVO treatment time was controlled to be 5 s (Figure 6a), all the electronic parameters of the OFETs were enhanced, including a positive shift of *V*_th_ (from -17.8 to -12.0 V), and an increasing *μ* (from 0.46 to 0.52 cm^2^/Vs). It was suggested that, at short UVO exposure time, although the chemical group on the PS dielectric surface would not change significantly, the surface of PS was effectively cleaned by UVO treatment [25]. As a result, the trap sites at the interface of pentacene/dielectric were reduced, leading to an improved performance [5,14]. However, when the UVO treatment time exceeded 10 s, all the parameters, except *V*_th_, started to become less along with the increasing UVO treatment time. An obvious decrease of *μ* (from 0.52 to 0.11 cm^2^/Vs) and the increase of SS (from 2.2 to 18.3 V/decade) were obtained. Furthermore, on/off ratio decreased about three orders of magnitude. As all the *I*_on_ were maintained at a relatively stable value of around 0.6 ~ 1 × 10^-4^ A (*I*_on_ was defined as the current of OFET when both *V*_gs_ and *V*_ds_ were -20 V), the decrease of on/off ratio could mainly be attributed to the increase of *I*_off_. To prove the universality of this approach, another group of OFETs based on C_60_ were fabricated [see Additional file 2]. The variation of all the parameters for C_60_ OFETs with different UVO treatment time exhibited a similar trend as those of the pentacene OFETs. It is worth noting that, with UVO treatment, n-type OFETs of C_60_ also exhibited a positive shift of *V*_th_, the increase of SS, and the decrease of *μ* and on/off ratio. However, unlike the OFETs consisting of p-type semiconductors, the decrease of on/off ratio was caused by the decrease of saturation current *I*_on_, rather than the increase of *I*_off_. The above results indicated that the surface property of PS dielectric was effectively modified by UVO treatment, and such modification could be well controlled by adjusting the UVO treatment time.

**Figure 7:**
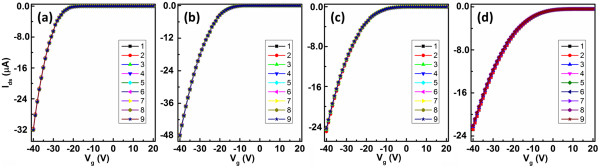
**Bias-stress characteristics of OFETs.** Bias-stress characteristics of OFETs with a typical **(a)** 0-, **(b)** 5-, **(c)** 60-, **(d)** 180-s UVO-treated PS.

The C-O, C = O, and O-C = O groups on the surface of the dielectric layer were regarded as charge trap sites and could influence the device performance accordingly [5]. Since the charge transport process is believed to occur within the very first few semiconducting layers in proximity to the gate dielectric, such trap sites are important in organic semiconductor-based OFETs [28]. The additional oxygen groups would act as an electron acceptor under depletion condition and induced the mobile hole charge carriers at the semiconductor/dielectric interface for charge balance. Thus, positively shifted *V*_th_ and increasing *I*_off_ were obtained. While under accumulation condition, the oxygen groups would act as the hole trap sites, which would reduce the mobility, and enlarge the SS, accordingly [29-31]. Moreover, the inferior morphology of the pentacene grown on long time UVO-exposed PS dielectric was also responsible for the degradation of OFET performance.Furthermore, to investigate if UVO treatment in the device is a long term modification, the transfer characteristics of OFETs with a typical 0-, 5-, 60-, 180-s UVO-treated PS were tested for nine times to elucidate the bias-stress effect, as shown in Figure 7. No obvious variation could be observed from the curves, indicating that the modification of UVO treatment to PS dielectric is stable and robust.

## Conclusions

The effect of UVO treatment on PS dielectric for pentacene OFETs was investigated, and the property of PS dielectric was analyzed through AFM and XPS. It was found that the surface property of PS dielectric could be quantitatively modified by adjusting the UVO treatment time. Consequently, the electronic properties of OFETs based on UVO-treated PS could be modulated. Enhanced performance with hole mobility up to 0.52 cm^2^/Vs and reduced *V*_th_ of -12 V was obtained in OFETs based on 5-s UVO-treated PS dielectric. These results indicated that UVO-treated PS dielectric holds the potential to regulate the performance of OFETs based on such dielectric, and thus, paves a new simple way to accelerate the development of nanoscale organic electronic devices.

## Competing interests

The authors declare that they have no competing interests.

## Authors’ contributions

WH carried out the design of the experiment and film characterization. HF and XZ mainly made contribution on performing the experiment and data analysis. JY is the supervisor of WH and the corresponding author of this work. All authors read and approved the final manuscript.

## Supplementary Material

Additional file 1 Output curves of OFETs consisting of PS dielectrics with a typical UVO treatment of (a) 0, (b) 5, (c) 60, and (d) 180 s. Click here for file

Additional file 2 **Transfer curves of OFETs based on C**_**60 **_**with UVO-treated PS dielectrics.** Click here for file
