# Supplementary figures and images for: Effect of UV/ozone treatment on polystyrene dielectric and its application on organic field-effect transistors
Source: Nanoscale Res Lett. 2014 Sep 10;9(1):479. doi: 10.1186/1556-276X-9-479 (PMC4164320; doi:10.1186/1556-276X-9-479)

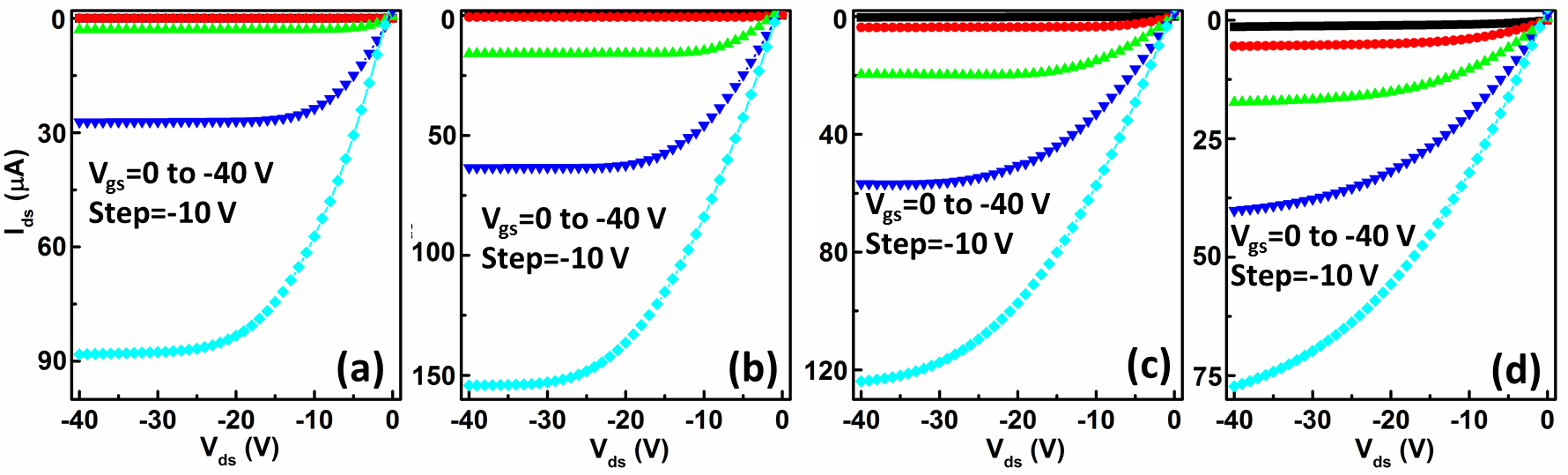

Supplement: Additional file 1 — Output curves of OFETs consisting of PS dielectrics with a typical UVO treatment of (a) 0, (b) 5, (c) 60, and (d) 180 s. [file 1556-276X-9-479-S1.tiff]

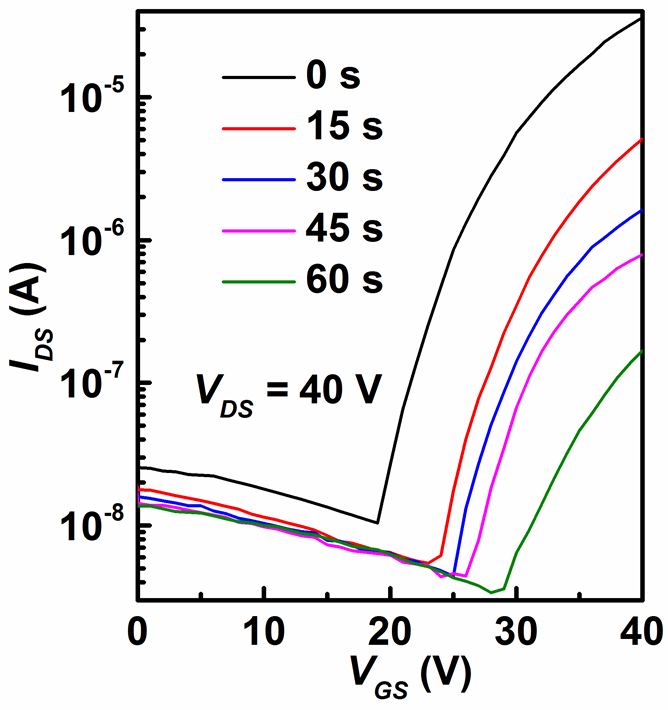

Supplement: Additional file 2 — Transfer curves of OFETs based on C60 with UVO-treated PS dielectrics. [file 1556-276X-9-479-S2.tiff]
